# Development of the Clinical Teaching Effectiveness Questionnaire in the United States

**DOI:** 10.3352/jeehp.2017.14.14

**Published:** 2017-06-29

**Authors:** Michelle E. Wormley, Wendy Romney, Anna E. Greer

**Affiliations:** Department of Physical Therapy and Human Movement Science, Sacred Heart University, Fairfield, CT, USA; Hallym University, Korea

**Keywords:** Physical therapists, Perception, Teaching, Surveys and questionnaires, Principal Component Analysis

## Abstract

**Purpose:**

The purpose of this study was to develop a valid measure for assessing clinical teaching effectiveness within the field of physical therapy.

**Methods:**

The Clinical Teaching Effectiveness Questionnaire (CTEQ) was developed via a 4-stage process, including (1) initial content development, (2) content analysis with 8 clinical instructors with over 5 years of clinical teaching experience, (3) pilot testing with 205 clinical instructors from 2 universities in the Northeast of the United States, and (4) psychometric evaluation, including principal component analysis.

**Results:**

The scale development process resulted in a 30-item questionnaire with 4 sections that relate to clinical teaching: learning experiences, learning environment, communication, and evaluation.

**Conclusion:**

The CTEQ provides a preliminary valid measure for assessing clinical teaching effectiveness in physical therapy practice.

## Introduction

Effective clinical instructors (CIs) are essential to the development of entry-level physical therapists. Doctoral physical therapy (DPT) programs require a minimum of 30 weeks of full-time clinical education (CE) experience led by CIs, in accordance with the Commission on Accreditation in Physical Therapy Education [1]. This emphasis on CE has led to the development of tools for assessing the clinical teaching effectiveness (CTE) of CIs. The majority of the literature, however, has focused on student perceptions of the CTE of CIs, applied qualitative methods, compared CTE among American Physical Therapy Association (APTA) credentialed versus non-credentialed CIs, and/or used validated tools from nursing [2-6].

Teaching characteristics associated with positive student outcomes in the physical therapy (PT) literature include communication, professionalism, interprofessional relations, teaching, and the ability to provide feedback [2-6]. However, a recent systematic review, highlighted the lack of reliable outcome measures and the heterogeneity of tools used to assess CTE [7]. For example, Morren et al. [3] used the 21-item section pertaining to ‘PT student assessment of clinical instruction’ of the APTA’s ‘Physical therapist student evaluation: clinical experience and clinical instruction’ survey to obtain information regarding CI characteristics and students’ perceptions of CI skills in relationship to APTA-credentialed versus non-credentialed CIs. The tool was reviewed for content validity by a CE special interest group of the APTA during survey development, but no other psychometric properties were investigated. Wetherbee et al. [6] also researched the teaching behaviors of APTA-credentialed versus noncredentialed CIs, but used an adapted version of the Nursing Clinical Teacher Effectiveness Inventory, a student assessment of nursing clinical teachers. The original version of the 47-item tool was found to be reliable and valid for measuring clinical teacher effectiveness; although the authors only examined internal consistency for use with PT students, they found the data collected by the tool to be reliable. Housel and Gandy [2] used demographic data and 27 items pertaining to CI-specific criteria of the NEC-ACCE (New England Consortium of Academic Coordinators of Clinical Education) ‘Student’s evaluation of CE experience.’ The tool was reviewed for face and content validity only and not for use with CIs. Buccieri et al. [4] used a qualitative grounded theory approach via semi-structured interviews to find meaning in CIs’ descriptions of clinical teaching. To date, a valid and reliable instrument to measure CTE from the CI’s perspective is lacking in PT. The purpose of this study was to develop a valid and reliable measure for assessing CTE in PT.

## Methods

### Study design

The Clinical Teaching Effectiveness Questionnaire (CTEQ) was developed via a 4-stage process: (1) initial content development, (2) content analysis by expert reviewers, (3) pilot testing, and (4) psychometric evaluation. This process, which followed recommended scale development procedures [8], is described below.

Investigators adapted a questionnaire to better capture the concepts of interest [9]. We used 5 of the 58 items as presented, revised 23 items, and added 15 items. This resulted in a 43-item questionnaire with 4 sections: learning experiences (LExps), learning environment (LEnv), communication, and evaluation. Respondents rated their teaching behaviors from ‘strongly disagree’ to ‘strongly agree’ using a 5-point Likert scale.

Questions associated with LExps included the CI’s ability to write objectives, facilitate practice, and utilize a variety of teaching tools that span the cognitive, psychomotor, and affective domains. The LEnv section included questions on the CI’s ability to provide an environment that fosters professional and clinical development, while taking into consideration difficult and exceptional students. Content pertaining to communication involved the ability to provide constructive feedback, listen effectively, and request assistance from the academic institution. Lastly, the section of evaluation assessed CTE related to the CI’s ability to identify a problematic domain, intervene, document, implement a solution, and evaluate the effects.

Eight expert physical therapists with > 5 years of experience and service as a CI for > 5 students participated in content validation. The majority of the CIs (83%) had obtained the American Board of Physical Therapy Specialty Certification, completed the basic CI training, held a teaching role in an accredited PT program, and had earned a DPT degree. CIs were provided information regarding the purpose of the questionnaire and score sheet to ensure a standardized review. They scored the question construction, interpretability, and relevance, and provided feedback on its comprehensiveness. The questionnaire was revised, and 1 item was eliminated, 10 were modified, and 10 were added.

### Subjects

For pilot testing, CIs from both universities were purposively sampled and requested to complete the questionnaire. The CI databases yielded 1,001 potential respondents, who were recruited via e-mail. The questionnaire and 2 follow-up reminders were sent [10]. Respondents consented by clicking a link that redirected them to the questionnaire, hosted on SurveyMonkey (San Mateo, CA, USA) (Table 1). A total of 205 CIs completed the questionnaire, a 20.5% response rate. The 205 respondents were mostly female (68.4%) with an average age of 40.54 years (standard deviation= 10.22 years). CI experience was distributed as follows: 1–5 years (19.1%), 6–10 years (47.4%), and 11+ years (33.5%).

Principal component analysis (PCA) was performed to refine the questionnaire, determine preliminary factorial validity, and identify potential scales within the 4 sections. Once the scales were identified, tests of internal consistency were used to reduce the number of items within each scale to achieve parsimony.

### Technical information and statistics

Data were downloaded from SurveyMonkey into Excel (Microsoft Corp., Redmond, WA, USA) and loaded in PASW SPSS ver. 18.0 (SPSS Inc., Chicago, IL, USA) for analysis. Descriptive statistics were used for the sample. An exploratory analysis employing PCA was undertaken (with varimax rotation) to identify any factors within the 4 sections of LExps (12 items), LEnv (7 items), communication (9 items), and evaluation (15 items). Varimax rotation was used because our interest was in identifying whether the items developed for one construct were distinct from the items that would load on another construct. A loading of < 0.40 was employed as the cut-off. Any items exhibiting poor fit were eliminated. Poor fit was defined as any item that had a low communality score (< 0.40), did not load on any factor within a given section (< 0.40), or showed cross-loading on 2 factors (i.e., > 0.40 on more than 1 factor). PCA was re-run until a final factor solution was identified for each section. Next, tests of internal consistency utilizing the Cronbach alpha were calculated for each of the factors indicated by the PCA. The Cronbach alpha output indicating alpha values for the scale if items were deleted was used to reduce the number of items within each identified scale. Items were only removed if their removal would increase the alpha value or not cause a substantial reduction in the alpha value.

### Ethical approval

This study was approved by the institutional review boards of Clarkson University (#76677) and Sacred Heart University (#150326A).

## Results

### Learning experiences

Preliminary analysis confirmed the factorability of the data for LExp (Kaiser-Mayer-Olkin [KMO]= 0.834; Bartlett test of sphericity, P< 0.001). Item 10 exhibited a low communality score (0.002) and was removed from the analysis. Raw data were available from Supplement 1. The PCA undertaken with the remaining items identified the presence of 2 components with eigenvalues exceeding 1.0, which we referred to as objective efficacy (items 3, 4, 5, and 6) and experience creation (items 1, 2, 7, 8, 9, 11, and 12). These components explained 35.80% and 15.43% of the variance, respectively, accounting for 51.22% of the total variance in LExp. The Cronbach alpha for the 4-item objective efficacy scale was 0.855. However, with item 6 removed, the Cronbach alpha increased to 0.861, resulting in a 3-item objective efficacy scale. All 3 of these items reflect a CI’s ability to write objectives for Lexp. The Cronbach alpha for the 7-item experience creation scale was 0.738. However, with item 8 removed, the Cronbach alpha decreased to 0.729, and with item 7 deleted it decreased to 0.718. All 5 items retained reflect a CI’s ability to create LExp for students. PCA was re-run using the reduced scales. The final solution yielded 2 factors (Table 2): objective efficacy (items 3, 4, and 5) and experience creation (items 1, 2, 9, 11, and 12) with eigenvalues exceeding 1.0, explaining 41.19% and 18.60% of the variance in LExp, respectively, thereby jointly accounting for 59.79% of the total variance in LExps.

### Learning environment

Preliminary analysis confirmed the factorability of the data for LEnv (KMO=0.820; Bartlett test of sphericity, P< 0.001). Item 18 had a low communality score (0.363) and was removed from the analysis. The PCA undertaken with the remaining items identified 1 factor (items 13, 14, 15, 16, 17, and 19) with an eigenvalue of 3.196, accounting for 52.27% of the total variance in LEnv. The Cronbach alpha for the 6-item LEnv scale was 0.817. However, with item 15 removed, the Cronbach alpha only decreased to 0.803. All 5 remaining items reflect a CI’s ability to create an effective LEnv through pedagogical methods. Additional PCA was not run since the final 5-item LEnv scale (items 13, 14, 16, 17, and 19) only represented 1 factor.

### Communication

Preliminary analysis confirmed the factorability of the data for communication (KMO=0.817; Bartlett test of sphericity, P<0.001). Item 25 exhibited a low communality score (0.398) and was removed from the analysis. Additionally, item 21 did not load on the factors and was removed. The PCA undertaken with the remaining items identified the presence of 2 factors with eigenvalues exceeding 1.0, which we referred to as feedback facilitation (items 20, 22, 23, and 24) and diverse communication (items 26, 27, and 28). Feedback facilitation and diverse communication explained 49.08% and 14.51%% of the variance, respectively, jointly accounting for 63.59% of the total variance.

The Cronbach alpha for the 4-item feedback facilitation scale was 0.818. However, with item 20 removed, the Cronbach alpha increased to 0.824, resulting in a 3-item feedback facilitation scale. All 3 items (22, 23, and 24) reflect a CI’s ability to provide and solicit student feedback. The Cronbach alpha for the 3-item diverse communication scale was 0.660. These 3 items (26, 27, and 28) reflect a CI’s ability to communicate with a diverse group of students including difficult and exceptional students. PCA was re-run using the reduced scales. The final solution yielded 2 factors (Table 3): feedback facilitation (items 22, 23, and 24) and diverse communication (items 26, 27, and 28) with eigenvalues exceeding 1.0, explaining 50.667% and 16.88% of the variance in communication, respectively, and jointly accounting for 67.54% of the total variance in communication.

### Evaluation

Preliminary analysis confirmed the factorability of the data for evaluation (KMO=0.831; Bartlett test of sphericity, P< 0.001). Item 42 exhibited a low communality score (0.368) and was removed from the analysis. Additionally, item 34 cross-loaded on 2 factors and was removed. The PCA undertaken with the remaining evaluation items identified the presence of 3 factors with eigenvalues exceeding 1.0, which we referred to as solution monitoring (items 36, 37, 38, 39, and 40), student assessment (items 29, 30, 35, 41, and 43), and domain identification (items 31, 32, and 33). Solution monitoring, student assessment, and domain identification explained 42.68%, 14.48%, and 9.94% of the variance, respectively, jointly accounting for 67.10% of the total variance.

The Cronbach alpha for the 5-item solution monitoring scale was 0.846. However, with item 36 removed, the Cronbach alpha increased to 0.873. With item 37 removed, the scale retained a Cronbach alpha of 0.847, resulting in a 3-item solution monitoring scale. All three items (38, 39, and 40) reflect a CI’s ability to monitor the effects of solutions implemented to address student learning issues. The Cronbach alpha for the 5-item student assessment scale was 0.811. No items were removed, as the Cronbach alpha would have dropped below 0.80 by doing so. All 5 items (29, 30, 35, 41, and 43) reflect a CI’s ability to assess student performance in clinical experiences. The Cronbach alpha for the 3-item domain identification scale was 0.912. All 3 items (31, 32, and 33) reflect a CI’s ability to identify the learning domains in which students are having difficulty. PCA was then re-run using the reduced scales. As shown in Table 4, the final solution yielded 3 factors with eigenvalues exceeding 1.0: solution monitoring (items 38, 39, and 40), student assessment (items 29, 30, 35, 41, and 43), and domain identification (items 31, 32, and 33). Solution monitoring, student assessment, and domain identification explained 43.80%, 16.77%, and 10.21% of the variance in evaluation, respectively, jointly accounting for 70.78% of the total variance.

## Discussion

The CTEQ has sound psychometric properties for the LExp, LEnv, communication, and evaluation sections. Content validity was achieved through a standardized review process by expert CIs. PCA was used to identify factors within each section. The Cronbach alpha was used to further reduce the number of items for each identified factor while retaining internal consistency reliability. Factorial validity was supported by the interpretability of the PCA results.

During the analysis, 4 items were removed from the original 12-item LExp section. The final solution for the LExp included 2 factors: objective efficacy and experience creation, both of which exhibited high reliability [11]. The objective efficacy scale examined a CI’s ability to establish clear teaching objectives. Setting clear goals and expectations for the students has been found to contribute to effective LExps. The experience creation scale captures CIs’ ability to create LExps across a variety of student learning styles and abilities. In prior studies, students reported that CIs who take the time to get to know the student’s preferred learning style, respond to the student’s individual learning needs, engage the student as an adult learner, and use multiple types of instructional strategies create an effective LExp [4,12,13]. Additionally, the PT literature has identified essential CI characteristics as including goal setting and goal writing, as well as teaching and learning styles [2].

The LEnv section originally included 7 items, but 2 were removed. The final 5 items represent a single factor: LEnv, which exhibited high reliability [11]. Creating a safe, supportive environment allowing for student questions, engaging in dialogue with positive regard, and encouraging the sharing of knowledge are measures that promote students’ attainment of knowledge and clinical decision-making skills [12,14]. PT education programs are required to perform ongoing assessments to evaluate clinical partnerships and the effectiveness of the LEnv, further emphasizing the importance of this aspect of the students’ experience [1].

The communication section originally included 9 items, but 4 were removed. The final 5 items represent 2 factors: feedback facilitation (high reliability) and diverse communication (moderate reliability) [11]. With regard to feedback facilitation, seeking student feedback on CI-student interactions and promoting student self-reflection during the clinical experience have been highlighted as essential CI characteristics [13,14]. Additionally, PT students have identified the ability to provide direct and immediate feedback as an effective teaching strategy. Diverse communication is also important, as the CI’s ability to adapt communication to meet the needs of students has been associated with successful student outcomes [2].

The evaluation section originally included 17 items, of which 4 were removed during the analysis. The final solution included 3 factors: solution monitoring, student assessment, and domain identification, all of which exhibited high reliability [11]. The ability to implement a multi-modal approach when developing assessment feedback (student assessment), to identify the areas or domains of strength and needed improvement (domain identification), and to provide both formative and summative feedback (solution monitoring) have been noted as effective clinical teaching strategies [9].

The CTEQ was developed with a cohort of CIs from 2 universities in the Northeastern region of the USA. Additional research should examine the utility and psychometric properties of this instrument with additional cohorts of physical therapists from across the US. The questionnaire should also be examined with CIs from other healthcare professions to determine whether the instrument is applicable. The identified subscales are consistent with the literature; however, future research using confirmatory factor analysis (CFA) is needed to validate the subscales identified using PCA in this study.

Study findings suggest that a 30-item measure is valid with a sample of CIs for PT programs in the Northeast with the following sections and subscales: LExp (objective efficacy and experience creation), LEnv, Communication (feedback facilitation and diverse communication), and evaluation (solution monitoring, student assessment, and domain identification). In its current format, the measure displays acceptable reliability and validity, and is suitable for administration as a measure of CTE in PT.

## Figures and Tables

**Table 1.** Clinical Teaching Effectiveness Questionnaire sections, subscales, item numbers, and items

Section	Subscale	Item #	Item
Learning experiences	Experience creation	1	I plan learning experiences for the student based on behavioral objectives and overall objectives for the clinical experience.
	Experience creation	2	I plan learning experiences that challenge the student and clinical instructor's preferred learning/teaching styles.
	Objective efficacy	3	I write individual behavioral objectives for learning experiences in the cognitive domain.
	Objective efficacy	4	I write individual behavioral objectives for learning experiences in the psychomotor domain.
	Objective efficacy	5	I write individual behavioral objectives for learning experiences in the affective domain.
		6^a)^	I write behavioral objectives that clearly describe expectations of the student.
		7^a)^	I foster hands-on practice of a new skill.
		8^a)^	I use a variety of teaching tools such as patient simulation, role-play, or “paper patients” to enhance each students' learning.
	Experience creation	9	I match the learning experiences and opportunities with the student's learning needs.
		10^a)^	I do not expect the student to collaborate on weekly goal planning.
	Experience creation	11	I am effective at individualizing and tailoring learning experiences for the difficult student.
	Experience creation	12	I am effective at individualizing and tailoring learning experiences for the exceptional student.
Learning environment	Learning environment	13	I consciously provide a learning environment that fosters the student's development of clinical skills.
	Learning environment	14	I consciously provide a learning environment that fosters the student's professional development.
		15^a)^	I consciously demonstrate behaviors consistent with core values of professionalism in my daily practice (accountability, altruism, compassion/caring, excellence, integrity, professional duty, and social responsibility).
	Learning environment	16	I use high level questioning to apply knowledge to decision making.
	Learning environment	17	I expect the student to provide evidence to support their clinical decision making.
		18^a)^	I am effective at creating a learning environment for the difficult student.
	Learning environment	19	I am effective at creating a learning environment for the exceptional student.
Communication		20^a)^	I facilitate communication with the student through active listening.
		21^a)^	I avoid communication that may be difficult or confrontational with the student.
	Feedback facilitation	22	I give timely feedback during the clinical experience to further learning and/or modify behavior.
	Feedback facilitation	23	I give constructive feedback during the clinical experience to further learning and/or modify behavior.
	Feedback facilitation	24	I expect students to seek ongoing feedback even if it is not required by the school.
		25^a)^	I request assistance from the center coordinator of clinical education, in my facility, as needed for problem solving.
	Diverse communication	26	I communicate with the academic coordinators of clinical education/director of clinical education from the school regarding student performance (positive and negative).
	Diverse communication	27	I am effective at communicating with the difficult student.
	Diverse communication	28	I am effective at communicating with the exceptional student.
Evaluation	Student assessment	29	I carefully observe the student to determine his/her individual strengths and areas to develop.
	Student assessment	30	My student evaluations are based on first-hand information.
	Domain identification	31	I am able to identify the cognitive domain in which the student is having difficulty.
	Domain identification	32	I am able to identify the affective domain in which the student is having difficulty.
	Domain identification	33	I am able to identify the psychomotor domain in which the student is having difficulty.
		34^a)^	I document change in the student's performance of behavior.
	Student assessment	35	I address problems as they arise with the student.
		36^a)^	I document the strategies I implemented to remediate the problem.
		37^a)^	I am effective at evaluating the effects of the implemented sdution for the difficult student.
	Solution monitoring	38	I am effective at evaluating the effects of the implemented sdution for the exceptional student.
	Solution monitoring	39	I am effective at modifying the sdution to meet the needs of the student with difficulties.
	Solution monitoring	40	I am effective at modifying the sdution to meet the needs of the exceptional student.
	Student assessment	41	I do not let my personal biases affect my evaluation of the student.
		42^a)^	I engage the student in self-assessment as part of analyzing performance.
	Student assessment	43	I consider all student factors (i.e., current level of performance, academic curriculum, level of didactic preparation) in analyzing his/her behavior.

a) Items included in the original questionnaire but recommended for removal in the final questionnaire.

**Table 2.** Learning experiences subscales: factor and reliability analysis

Item #	Factors	
	Objective efficacy	Experience creation
3	0.893	
4	0.867	
5	0.824	
1		0.699
2		0.690
9		0.685
11		0.682
12		0.602
Eigenvalue	3.303	1.408
Variance explained	41.19%	18.60%
Cronbach alpha	0.861	0.718

**Table 3.** Communication subscales: factor and reliability analysis

Item #	Factors	
	Feedback facilitation	Diverse communication
26		0.839
27		0.782
28		0.592
22	0.871	
23	0.860	
24	0.732	
Eigenvalue	3.040	1.013
Variance explained	50.667	16.876
Cronbach alpha	0.824	0.660

**Table 4.** Evaluation subscales: factor and reliability analysis

Item #	Factors				
	Solution monitoring	Student assessment	Domain identification	
38	0.758		
39	0.825		
40	0.826		
29		0.775	
30		0.763	
35		0.715	
41		0.622	
43		0.718	
31			0.894
32			0.880
33			0.894
Eigenvalue	4.818	1.844	1.124
Variance explained	43.80%	16.77%	10.21%
Cronbach alpha	0.847	0.811	0.912

## Supplementary materials

Supplement 1. Data file is available from https://dataverse.harvard.edu/dataset.xhtml?persistentId=doi%3A10.7910%2FDVN%2F7QPCVV

Supplement 2. Audio recording of the abstract.
